# Photo‐Induced Stepwise Liquid Crystal Phase Transition Enabled by Hydrazone Photoswitch

**DOI:** 10.1002/advs.78402

**Published:** 2026-10-22

**Authors:** Shijie Liu, Yuxin Zhou, Junwen Chen, Yexin Wang, Yiqiu Lv, Panjie Wang, Wenqi Yu, Gege Meng, Jiayi Yu, Chunhua Lin, Guo‐Hua Hu, Ruochen Lan

**Affiliations:** ^1^ Jiangxi Province Key Laboratory of Porous Functional Materials School of Chemical Engineering Jiangxi Normal University Nanchang People's Republic of China; ^2^ CNRS LRGP Université De Lorraine Nancy France

**Keywords:** linear‐shaped hydrazone, liquid crystals, phase transition temperature, photo‐induced stepwise phase transition

## Abstract

Advanced liquid crystal (LC) materials with photo‐induced phase transition behaviors hold significant scientific and technological importance because of its potentials in next‐generation optoelectronic devices and information encryption. Azobenzene‐based LC has been widely studied in this field for several decades. However, the relatively poor thermal stability of the *cis* isomer of azobenzene severely limits its applications in state‐locking and high‐temperature scenarios. Herein, a photoisomerizable and bistable hydrazone molecule was judiciously designed and synthesized. The hydrazone photoswitch undergoes linear‐to‐bent configurational changes via light‐driven isomerization, achieving isothermal and reversible control of LCs from ordered to disordered states. The perturbation effects of hydrazone isomerization on LC phase were analyzed by measuring the dichroism ratio (***R***) and order parameter (***S_Φ_***). Thanks to the bistability of hydrazone molecules, the phase transition temperatures of the LCs can be precisely and stepwise controlled by regulating light intensity and exposure time. Utilizing the unique light‐induced phase transition of hydrazone‐doped cholesteric liquid crystals (CLCs), thermoresponsive colorful optical devices with reversibly writable and erasable patterns by light were demonstrated, highlighting their significant potential for applications in smart optical materials.

## Graphical abstract

A judiciously designed bistable hydrazone photoswitch enables a photo‐induced stepwise “order‐disorder” phase transition in liquid crystals. The linear‐to‐bent isomerization disrupts molecular packing, allowing precise tuning of phase transition temperatures. By doping this switch into cholesteric liquid crystals, thermoresponsive optical devices with reversibly writable and erasable structural color patterns are demonstrated, offering significant potential for information encryption.

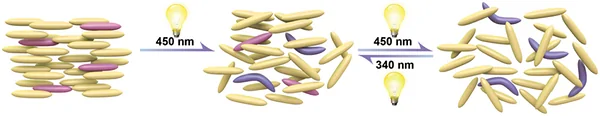

## 1 Introduction

Stimuli‐responsive phase transition of liquid crystals (LCs) has attracted intense scientific interest in the past decades because it not only provides insights into non‐equilibrium phase dynamics and soft matter physics but also opens avenues for soft actuators, tunable metasurfaces, and advanced photonic applications [1, 2, 3, 4, 5]. By utilizing light as an external stimulus, a remote, spatiotemporally precise, and non‐invasive control over the molecular ordering and macroscopic properties of LCs can be realized [6, 7, 8]. Advanced LC materials with photo‐induced phase transition features are essential for next‐generation optoelectronic devices, information encryption, and biomedical microsystems, bridging the fundamental soft matter research with cutting‐edge photonic engineering.

Photo‐induced phase transitions of LCs are generally driven by configurational changes of photoswitches, which can be categorized as “order‐disorder” [1] transition and “order‐order” transition [6] according to the orientational ordering changes of LCs. The former is comparably more fascinating and versatile, because this transition from an ordered (e.g., nematic or smectic) to a disordered (isotropic) state enables a large‐amplitude variation of the material's viscosity, birefringence, and molecular alignment, allowing the design and fabrication of optofluidic devices [9], adaptive optics [10, 11, 12], photoresponsive adhesives [13, 14], and soft actuators [15, 16, 17]. Azobenzene has been dominantly researched in this field. Upon UV light irradiation, azobenzene undergoes reversible *trans–cis* isomerization, which disrupts the molecular packing and reduces the orientational order of the LC phase [1, 18, 19, 20]. However, azobenzene suffers from the poor stability of its *cis‐*isomer and susceptibility to self‐heating relaxation, making long‐term phase stabilization challenging and severely limiting its applications in state‐locking scenarios. Developing novel photo‐active molecules with both disruptive effects and bistability may open a new avenue for fabricating photo‐responsive devices with unprecedented functionalities.

In recent years, hydrazone photoswitches have garnered widespread attention due to their excellent thermal stability, negative photochromism, and pronounced geometric changes [11, 12, 21, 22, 23, 24, 25]. Recent pioneering works have reported the incorporation of hydrazone photoswitches into LC materials to prepare light‐driven soft actuators and light‐responsive color‐changeable cholesteric LCs (CLCs) with extremely high thermal stability. The only report about the phase transition of LC triggered by a hydrazone photoswitch in 2018 is based on a chiral hydrazone with an isosorbide backbone, which enables an isothermal phase transition of 5CB from chiral nematic phase to undistorted smectic A (SmA) phase, achieving an “order‐order” transition [6]. If the hydrazone can induce an “order‐disorder” transition of LCs, it will be possible to induce a transformation of LCs by a bistable hydrazone and achieve unprecedented stepwise modulation of adaptive optics, soft actuators and information encryption devices. Thus, distinct from the “order‐order” transitions reported in earlier hydrazone‐containing liquid crystal systems, our work exploits a bistable hydrazone photoswitch to realize an “order‐disorder” phase transition, and further enables multistep tuning of the clearing temperature.

Herein, a series of novel bistable hydrazone photoswitches with a linear structure as a rigid core was judiciously designed and synthesized as shown in Scheme 1a, which is named as LH‐X (X represents the number of carbon atoms in its end flexible chains). The detailed synthetic routes and NMR characterization data for all intermediates and final products are provided in Figures S1–S24. The single‐crystal structure and photo‐responsive behaviors of the LH‐X photoswitch were characterized. By doping the LH‐X molecules into commercially available LCs including 5CB, 7CB and 6OCB, a photo‐induced isothermal and reversible nematic to isotropic phase transition of the LC was observed (Scheme 1b). The mechanism of the photo‐induced “order‐disorder” transition of the LC was investigated by monitoring the dichroism ratio (***R***) and order parameter (***S_Φ_***) of the LC. Further molecular dynamics simulations confirm the orientational disordering of LC molecules after isomerization of LH‐X photoswitches, from the aspects of interaction energy and molecular rotational radius. The disrupting effect causes a decrease of nematic to isotropic phase transition temperature (T_NI_) of LCs, which can be accurately characterized by heating and cooling cycles due to the bistability of hydrazones. Finally, the application potential of this novel photo‐responsive LC material was demonstrated by doping the LH‐6 molecules into CLC. By spatially regulating the cholesteric‐to‐isotropic phase transition by light, arbitrary structural color patterns can be written and erased reversibly.

**SCHEME 1:**
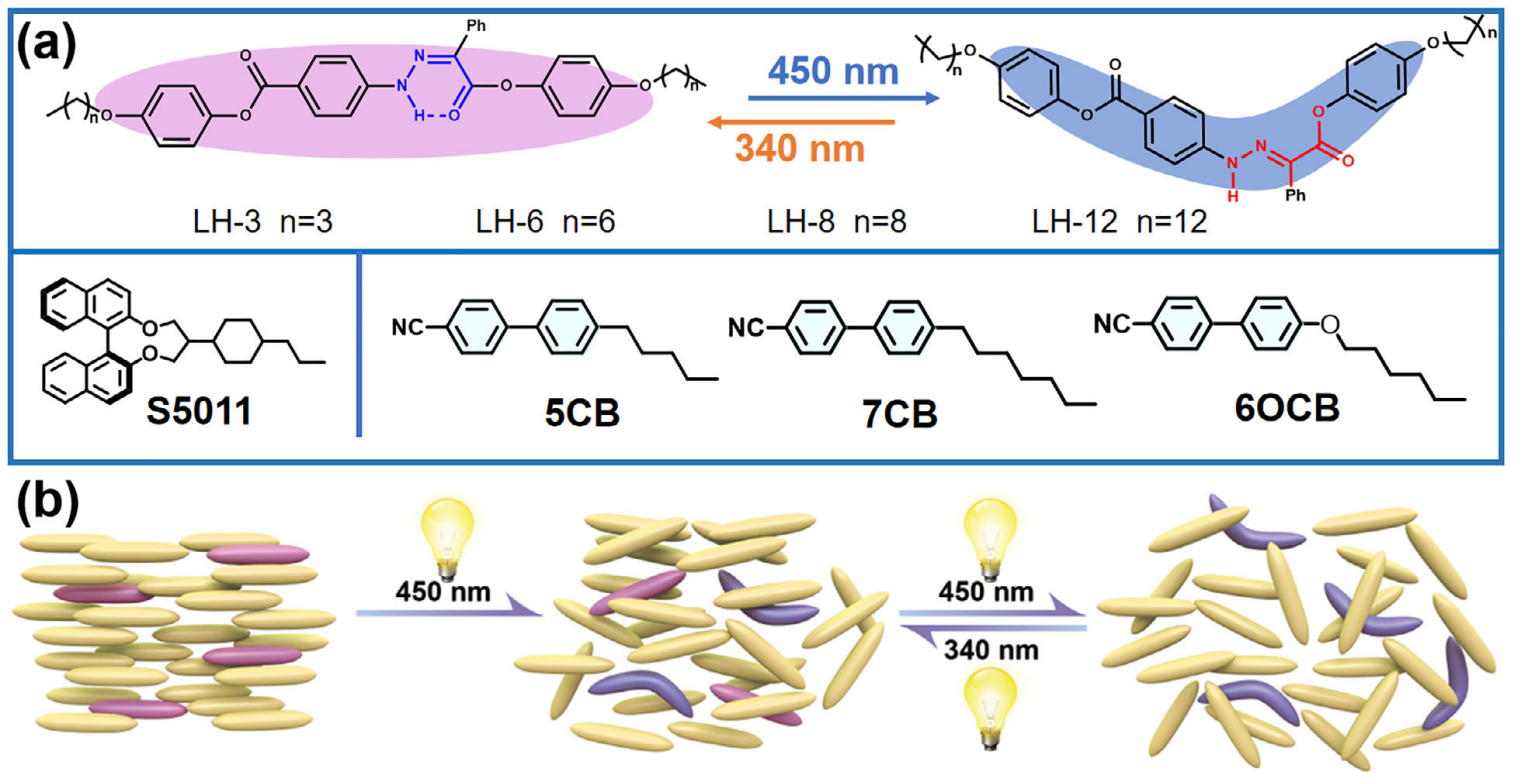
(a) *Z/E* isomerization of the light‐induced bistable hydrazone photoswitch, showing the molecular structures of the chiral molecule S5011 and the liquid crystal matrices 5CB, 7CB, and 6OCB. (b) Schematic illustration of photo‐induced phase transition. The LC (yellow) is doped by the hydrazone photoswitch (pink). The degree of disorder is determined by the quantity of *E*‐isomers (blue), which induce the nematic‐to‐isotropic phase transition.

## 2 Results and Discussion

### 2.1 Photo‐Responsive Behavior of LH‐X Photoswitch

The synthetic route of LH‐X was illustrated in the Supporting Information. Taking LH‐6 as an example, the photo‐responsiveness of LH‐6 was first investigated by dissolving it in solution. The spectral characteristics of the novel bistable photo‐responsive hydrazone switch at different photostationary states (PSSs) were examined using UV–vis absorption spectroscopy. As shown in Figure 1a, LH‐6 was dissolved in THF with a concentration of 10 µm. Irradiation the solution with 450 nm light (2 mW·cm^−^^2^, 3 min), resulting in a blue‐shift of the maximum absorption peak from 375 nm to 351 nm, indicating the *Z‐E* isomerization of LH‐6 to the PSS under 450 nm light (PSS_450_). Subsequent irradiation with 340 nm light (0.1 mW·cm^−^^2^, 5 min) causes the absorption peak to red‐shift to 372 nm, achieving the PSS under 340 nm light (PSS_340_). The isomerization of LH‐6 at different PSSs was quantitatively investigated via ^1^H NMR spectroscopy. As illustrated in Figure 1b, under 450 nm light irradiation, the NMR signal corresponding to the *Z*‐isomer weakens and then completely disappears when it reaches PSS_450_, while a new signal associated with the *E*‐isomer occurs. Following 340 nm irradiation makes the *Z*‐isomer‐related signal reappear, yielding a *Z: E* ratio of 83:17 at PSS_340_. The cycling tests of photoisomerization of LH‐6 were conducted by monitoring the absorbance of LH‐6 solution at 400 nm. There was no fatigue after ten cycling tests (Figure 1c). The photochemical properties of LH‐3, LH‐8, and LH‐12 molecules were presented in Figures S31–S33 and S35–S40, which are similar to those of LH‐6. The LH‐6 photoswitch is bistable with extremely high thermal stability; the measured half‐life time of the *E*‐isomer of LH‐6 is near 30 years (Table S1). Based on the kinetic data (Figures S43–S50), the quantum yields of LH‐X were measured. Specifically, the quantum yields of LH‐6 were determined to be Φ_Z→E_ = 29.82% and Φ_E→Z_ = 8.43%, with the detailed data summarized in Table S2.

**FIGURE 1:**
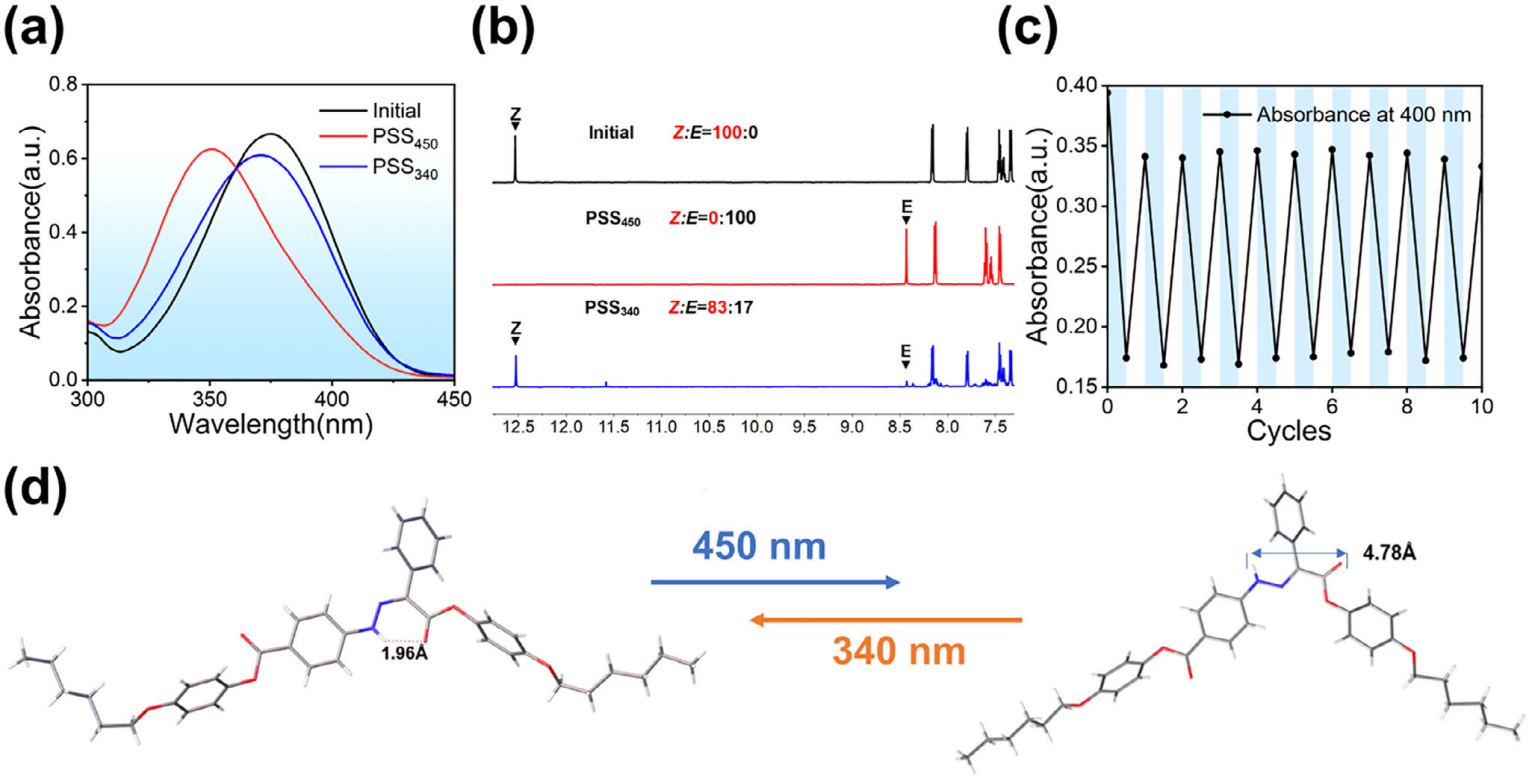
(a) Absorption spectra of the LH‐6 molecules in THF with a concentration of 10 µM at different states. (b) ^1^H NMR spectra of the LH‐6 molecules under different PSSs. (c) Absorbance changes of the LH‐6 solution at 400 nm during cycling photoirradiation. (d) Crystal structures of the *Z*‐isomer and *E*‐isomer of LH‐6 molecules.

The molecular structure of LH‐6 was analyzed by characterizing the single crystal structures of the *Z*‐isomer and the *E*‐isomer. Although the diffraction quality was not optimal, it did not affect the determination of the molecular structure and conformation. As shown in Figure 1d, the *Z*‐isomer possesses intramolecular hydrogen bonds between N‐H groups and adjacent carbonyl oxygen atoms (C═O), significantly enhancing molecular rigidity and preventing system disorder caused by free rotation of single bonds. Upon 450 nm light irradiation, the *Z*‐isomer converts to the *E*‐isomer, and the intramolecular hydrogen bond breaks. The distance of the corresponding H and O atoms increases from 1.96 to 4.87 Å. The *E*‐isomer presents a V‐shaped configuration with the breakage of intramolecular hydrogen bonds.

### 2.2 Photo‐Induced Phase Transition of LH‐Doped LC

The LH‐6 was then doped into the LC matrix 5CB. The photo‐responsive properties of LC were investigated by polarized optical microscopy (POM). At 30°C, the LC presents a typical aligned nematic phase texture. By exposing the sample to 450 nm light, it transitions to the isotropic phase. The phase transition is reversible by following 340 nm light irradiation (Figure 2a). The isomerization of LH‐6 was monitored during this process by UV–vis spectra, indicating the capability of LH‐6 to induce the “order‐disorder” phase transition (Figure 2b). This is evidenced by the gradual blue‐shift of the absorption maximum from 387 to 350 upon 450 nm irradiation (*Z*‐to‐*E* isomerization) and the reversible red‐shift back to 380 upon 340 nm irradiation (*E*‐to‐*Z* isomerization). While these spectral shifts provide qualitative evidence for the photoisomerization, the exact PSS composition in the liquid crystal matrix remains uncertain due to the complex matrix environment and the lack of a validated calibration method.

**FIGURE 2:**
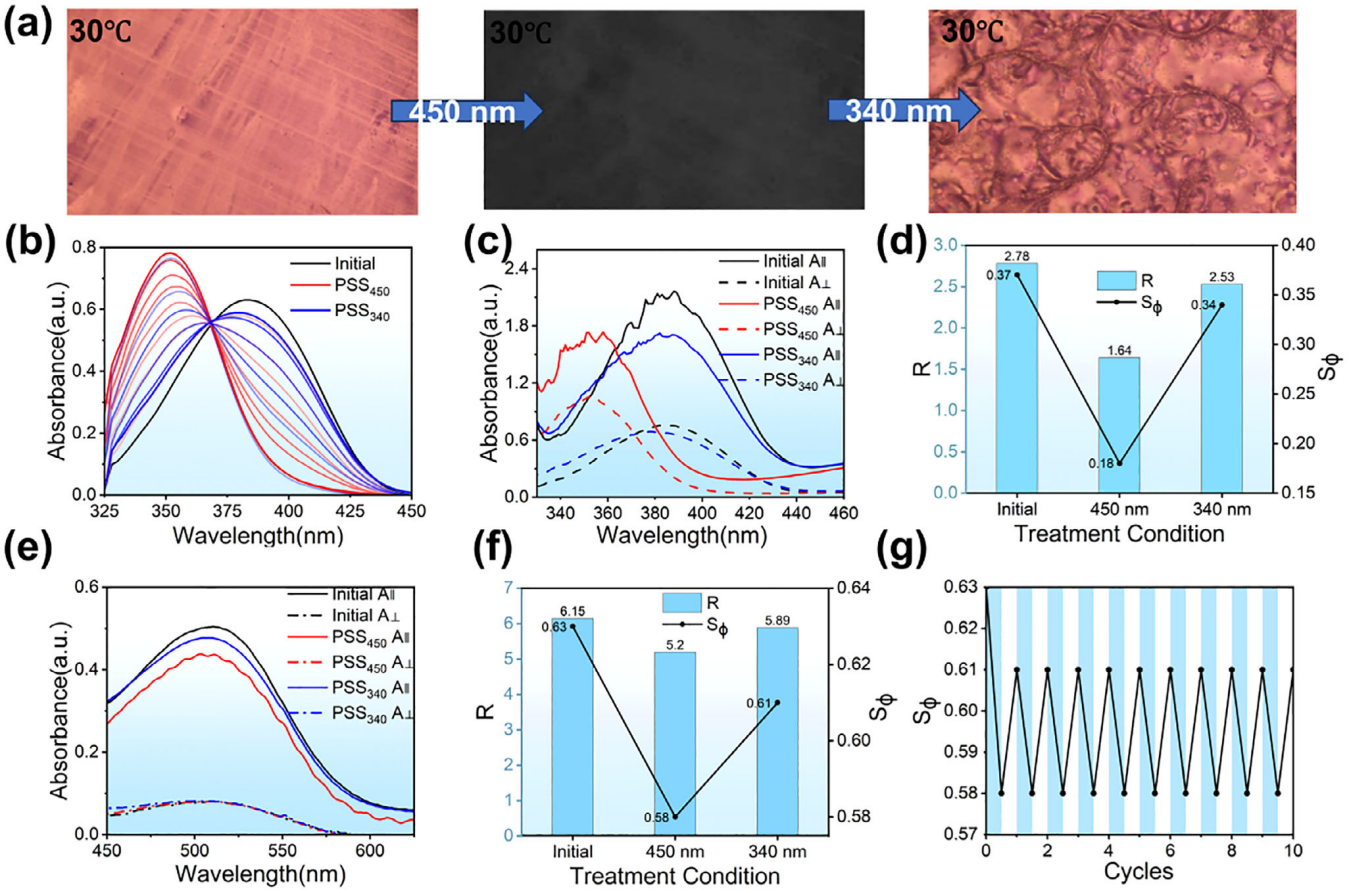
(a) A mixture of hydrazone LH‐6 (4 wt.%) and 5CB undergoes reversible photoswitching between the nematic phase and the isotropic phase upon irradiation with 450 and 340 nm light at 30°C. (b) UV–vis absorption spectra of the LH‐6 switch at 450 and 340 nm light irradiation (solvent: LC 5CB, concentration: 1 wt.%). (c) Linearly polarized absorption spectra of hydrazone LH‐6 under different states. (d) ***R*** and ***S_Φ_*** of hydrazone LH‐6 under different states. (e) Linearly polarized absorption spectra of DR1 in LH‐6‐doped 5CB under different states. (f) ***R*** and ***S_Φ_*** of DR1 under different states. (g) Cycling test of ***S_Φ_*** of DR1 under alternating irradiation of 450 and 340 nm light.

To reveal the mechanism of Photo‐induced phase transition of LCs, two critical parameters: dichroism ratio ***R*** and order parameter ***S_Φ_*** were recorded at different states [26, 27, 28, 29, 30]. In this test, a small amount of LH‐6 (1 wt.%) was doped into 5CB. The mixture was filled into a planar‐aligned LC cell. Linearly polarized light absorption spectra of LH‐6 were collected to detect the orientation of LH‐6 in LCs. ***R*** is the ratio of absorbance at two orthogonal polarization directions, which is defined as:

$$R=\frac{A∥}{A⊥}$$

In which, A∥ and A⊥ are the absorbance along the director of the LC and along the perpendicular direction of the LC director, respectively. ***S_Φ_*** is used to evaluate the orientational ordering of LC molecules. The order parameter is defined as:

$$S_{\Phi }=\frac{R-1}{R+2}=\frac{A∥-A⊥}{A∥+2A⊥}$$

As shown in Figure 2b, the maximum absorption peak of the sample gradually shifts from 387 nm to 350 nm. Subsequent irradiation with 340 nm light causes the peak to redshift to 380 nm, confirming efficient photo‐isomerization of LH‐6 molecules in the LC matrix. The initial ***R*** of LH‐6 is 2.78, indicating that the LH‐6 molecule aligns along the director of the LC. After exposing the sample to 450 nm light, the dichroism ratio decreases from 2.78 to 1.64, and the ***S_Φ_*** decreases from 0.37 to 0.18. Subsequent exposure of the sample to 340 nm light enables ***R*** and ***S_Φ_*** to recover to 2.53 and 0.34, respectively. These results demonstrate that the orientation of the electronic transition moment of LH‐6 changes reversibly between different PSSs, which is similar with that in the previous report (Figure 2c,d) [6]. All of the other LH‐X molecules exhibit similar photo‐responsive ***R*** and ***S_Φ_*** changes (Figures S53–S55). The ***S_Φ_*** changes of LH‐6 molecules before and after isomerization cannot provide direct evidence for the photo‐induced phase transition of LC. To further illustrate the disrupting effect of LH‐6 in 5CB, Disperse Red 1 (DR1) was used as a molecular probe to detect the ordering of LC by mixing 0.5 wt.% DR1 into the LH‐6‐doped 5CB. As shown in Figure 2e, under 450 nm irradiation, DR1's A∥ at 506 nm slightly decreases, leading to a reduction in the ***S_Φ_*** from 0.63 to 0.58 (Figure 2f). The ***S_Φ_*** is totally reversible by alternative 450 and 340 nm light irradiation, indicating that the *E*‐isomer of LH‐6 exerts a more significant disturbing effect on the LC compared with that of the *Z*‐isomer (Figure 2g). The *Z‐E* isomerization lowers the order parameter of the LC, which plays a key role in the photo‐induced phase transition.

To investigate the interaction between the LH‐6 and LC molecules, we conducted molecular dynamics simulations using the Large‐scale Atomic/Molecular Massively Parallel Simulator (LAMMPS). Two models were adopted: the Z‐isomer‐doped LC and the E‐isomer‐doped LC. Energy minimization and annealing simulations were performed via LAMMPS (Figure 3a). After the system reached equilibrium, the radial distribution function, hydrogen bond count, interaction energy, radius of gyration, and order parameter were analyzed, with all values calculated from the final 1 ns of the production trajectory. It should be noted that, due to computational constraints, the reported results are derived from a single long‐time trajectory for each system, which limits the rigorous quantification of statistical uncertainty and convergence. Therefore, the following mechanistic interpretations are presented as qualitative observations rather than definitive statistical conclusions.

**FIGURE 3:**
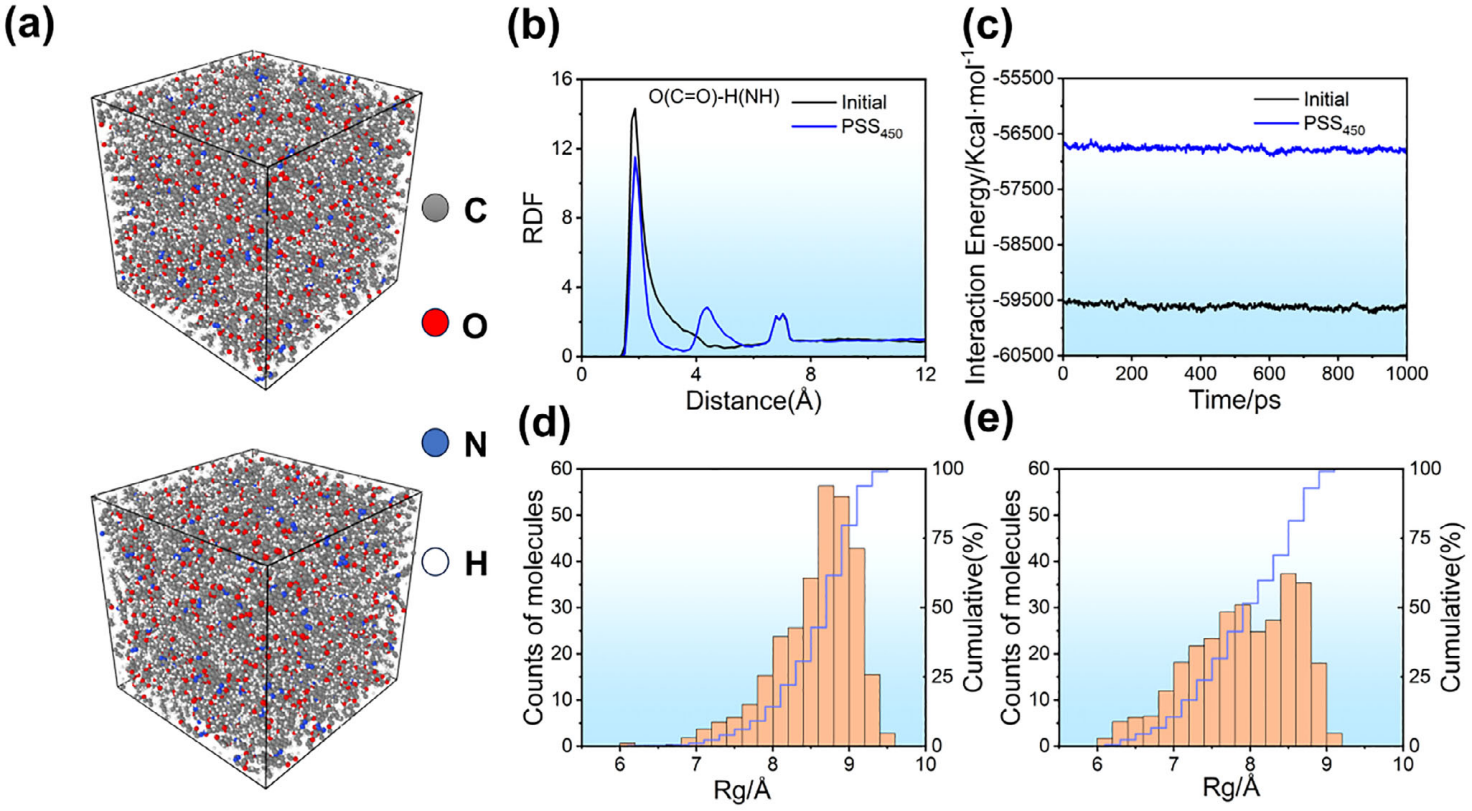
(a) Molecular dynamics simulation snapshots of the hydrazone photoswitch in its initial state (top) and PSS_450_ (bottom), following equilibrium with 5CB; gray represents C, red represents O, white represents H, and blue represents N. (b) Radial distribution functions of N‐H and carbonyl oxygen (C═O) before and after simulated isomerization. (c) Interaction energy between the hydrazone photo‐switch and 5CB before and after isomerization; lower energy values indicate stronger interactions and greater stability. (d) Rotation radius in the initial simulated state; (e) Rotation radius in PSS_450_.

In this single‐trajectory simulation, after isomerization of LH‐6, the peak at the characteristic distance of 2.0 Å exhibited a decreasing trend, suggesting that the short‐range ordered coordination structure associated with N‐H groups might be disrupted, which could potentially lead to the dissociation of hydrogen bond networks and a reduction in hydrogen bond count (Figure 3b). Concurrently, the absolute value of intermolecular interaction energy appeared to decrease, implying a possible weakening of intermolecular constraints and cohesive forces (Figure 3c). The molecular chain rotational radius showed a tendency to decrease, and the LH‐6 appeared to transition from an extended configuration to a bent configuration, which might reflect overall chain contraction (Figure 3d,e), further suggesting a reduction in the order parameter (Figure S57). Overall, based on these single‐trajectory observations, the photoisomerization process tends to disrupt the original ordered intermolecular coordination network, reducing both short‐range and long‐range order, thereby possibly driving the LC from an ordered to a disordered state. However, further validation using multiple independent trajectories is necessary to establish the statistical robustness of these mechanistic conclusions.

Then we measured the T_NI_ of LC before and after isomerization of LH‐6 by differential scanning calorimetry (DSC). Three commercially available LCs, 5CB, 7CB, and 6OCB, were used. The samples were prepared by doping 4 wt.% LH‐6 molecules into the LC matrix. Taking 5CB as an example, after three measurements and averaging, the sample presents a T_NI_ of 34.07 ± 0.17°C, which decreases to 28.00 ± 0.26°C after 450 nm light irradiation and then increases back to 32.01 ± 0.25°C upon 340 nm light irradiation (Figure 4a), the incomplete temperature recovery can be attributed to the incomplete *E*→*Z* photoisomerization of LH‐6 under PSS_340_ irradiation, a conclusion that is in agreement with the experimental results recorded in Figure 1b. The samples prepared from 7CB and 6OCB exhibit similar photo‐responsive changes of T_NI_ as shown in Figure 4b,c. Taking advantage of the bistability of the hydrazone photoswitch, a stepwise control of T_NI_ of LC was realized—a capability rarely achieved in azobenzene‐based LCs. As shown in Figure 4d–f, all of the three samples present a gradual decrease of T_NI_ under a constant 450 nm light field. To verify the post‐irradiation stability, we monitored the selected intermediate state in the dark after switching off the light. As shown in Figure S62, the T_NI_ remained highly stable at approximately 31.9 ± 0.2°C over 24 h. This directly confirms that the intermediate states can be retained after irradiation stops. The influence of light intensity on changes of T_NI_ was investigated. The T_NI_ change rates of all three samples increase with light intensity, and the control experiment on the blank LC host excludes photothermal effects, confirming that this enhancement arises from the accelerated LH‐6 isomerization at higher intensities(Figure S63). Under a 9 mW/cm^2^ 450 nm light, the T_NI_ decreases to the stationary state within 3 min (Figure 4g–i). The LH‐X molecules with different lengths of alkane chains present a similar capability to tune the T_NI_ of LC, and no clear linear trend is observed with increasing chain length. This observation indicates that, within the present molecular system, the length of the terminal flexible alkyl tail is not the dominant factor governing the photoinduced phase transition. Instead, the decisive structural parameter is the drastic shape change from a linear (*trans*) to a highly bent (*cis*) conformation upon UV irradiation. (Figures S58–S60 and Stable S3).

**FIGURE 4:**
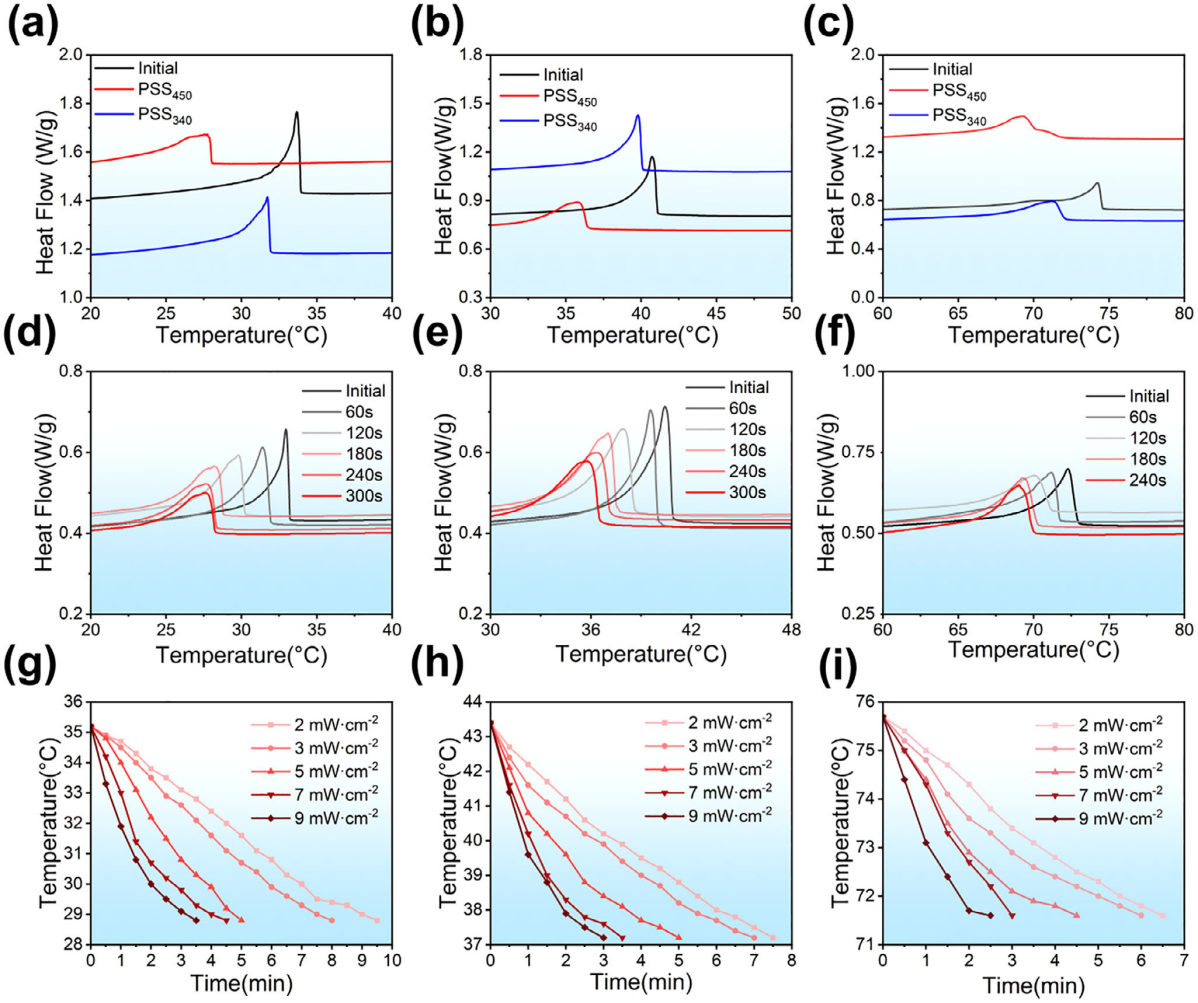
T_NI_ of (a) LH‐6‐doped 5CB, (b) LH‐6‐doped 7CB and (c) LH‐6‐doped 6OCB at different states. Stepwise T_NI_ of (d) LH‐6‐doped 5CB, (e) LH‐6‐doped 7CB, and (f) LH‐6‐doped 6OCB under a constant 450 nm light field. Changes of T_NI_ of (g) LH‐6 doped 5CB, (h) LH‐6 doped 7CB and (i) LH‐6 doped 6OCB at different light intensities.

To confirm the effectiveness of the linear structural design of the LH‐X photoswitch, a conventional bent‐shaped hydrazone (named BH‐6) with a similar end group was synthesized. The detailed synthetic routes and NMR characterization data for all intermediates and final products are provided in Figures S25–S30. Furthermore, its photochemical properties were evaluated for comparative analysis (Figures S34 and S41–S42). By doping the reference molecule BH‐6 into 5CB, there are no photo‐induced T_NI_ changes of LC after triggering the photoisomerization of BH‐6 (Figure S61). At the same time, the ***S_Φ_*** of the sample shows no changes after light irradiation, indicating that the perturbation effects and photo‐induced phase transition behavior can only be triggered by the linear structured LH‐X molecules judiciously designed in this work (Figure S56). In addition, based on the kinetic data (Figures S51 and S52), the quantum yields of BH‐6 were determined to be Φ_Z→E_ = 11.91% and Φ_E→Z_ = 1.63% (Table S2), which are significantly lower than those of LH‐6. This indicates that the bent geometry possesses a higher photochemical energy barrier and a slower response rate. It is worth noting that the PSS is established through a dynamic equilibrium between the forward and reverse photoreactions. Consequently, the PSS composition is determined by the ratio of the forward and reverse reaction rates, which depend on both the quantum yields and absorption cross‐sections. Therefore, the differences in quantum yields and absorption properties between LH‐6 and BH‐6 may lead to different exact PSS compositions. However, since all measurements in this work were performed after a stable PSS had been fully established, the observed differences in phase‐transition behavior are essentially attributable to the distinct modulation of liquid‐crystal assembly by the linear versus bent molecular geometry under their respective PSS conditions.

### 2.3 Photo‐Writable and Thermochromic CLC Device

Based on the light‐induced phase transition property, a chiral dopant S5011 was added to the photo‐responsive LC to make the phase transition to be visualized by the structural color change of CLC. The reflection colors of CLC can be precisely controlled by adjusting the doping concentration of S5011. When the concentration of S5011 increases from 2 to 3 wt.%, the reflection peak exhibits a blue shift, and the reflection color correspondingly changes from red to green and then to blue (Figure S64). As shown in Figure 5a, the phase transition of CLC was confirmed by POM observation. The sample initially presents a typical oily streak texture, which disappears after 450 nm light irradiation, indicating the CLC transitions to the isotropic phase. At the same time, the reflection color, as well as the reflection band of CLC, disappears after 450 nm light irradiation (Figure S64). The cholesteric texture and structural color of CLC can reoccur by a subsequent 340 nm light exposure due to the back isomerization of LH‐6. Taking advantage of the stepwise modulation of LH‐6‐doped CLC, the phase transition temperatures of different regions of a single device can be finely controlled, enabling multiple information encryption of the CLC with gradual change of temperature via color‐to‐colorless transitions of different regions. As shown in Figure 5b, subsequent slow heating of the sample with a red background color reveals the “rose” pattern at 29.4°C, while the “star” pattern becomes clearly visible at 31.2°C. When the temperature reached 36°C, the sample completely transitioned to an isotropic phase. By tailoring the content of S5011, the background color of the device can be finely tuned to green and blue, indicating a feasible approach to fabricate colorful displays with photo‐writable and thermo‐responsive patterns.

**FIGURE 5:**
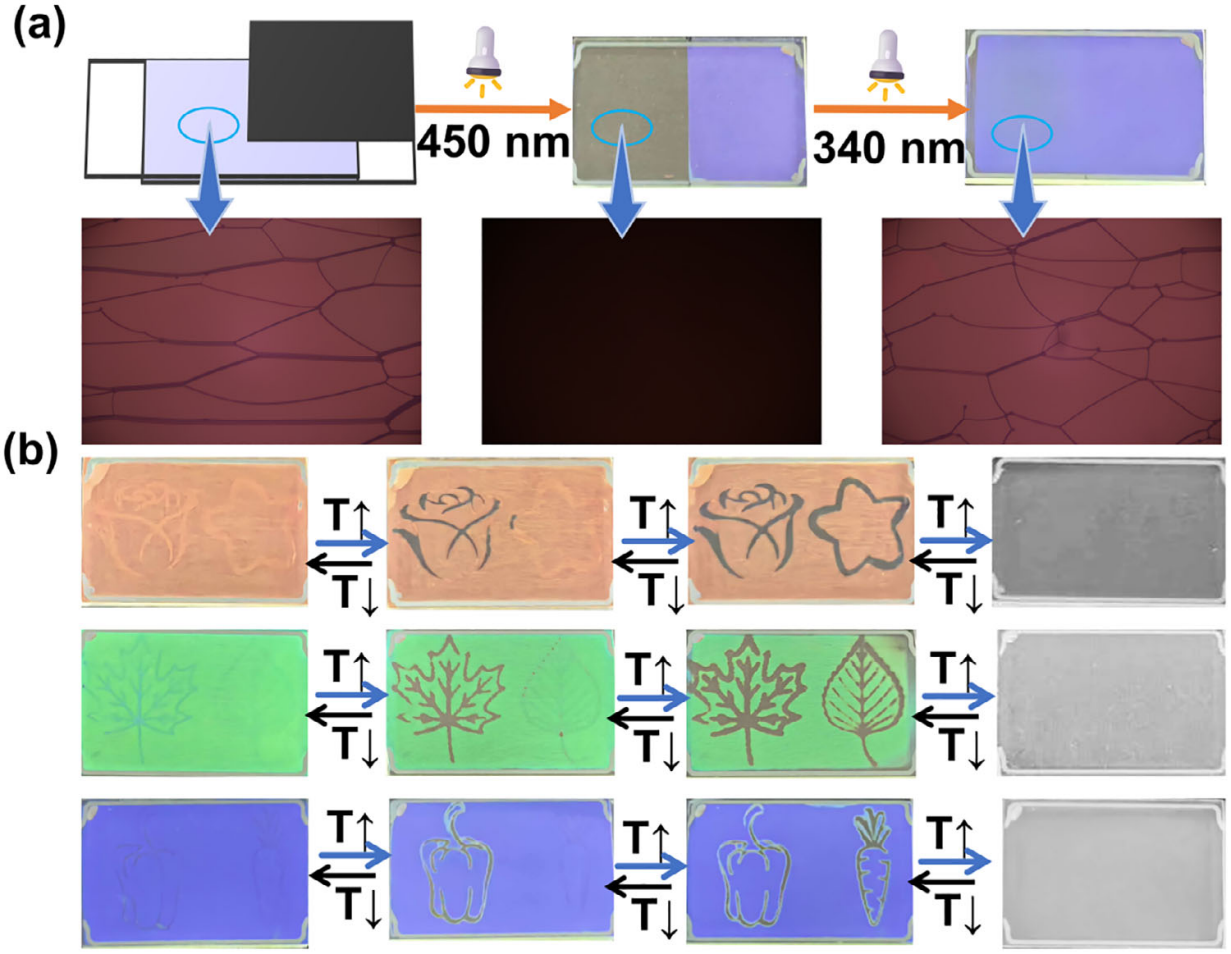
(a) Schematic illustration of the photo‐induced color‐to‐colorless transition of CLC and the corresponding POM images. (b) The sample was loaded with 2 wt.% (red), 2.5 wt.% (green), and 3 wt.% (blue) S5011 into the liquid crystal cell to form a CLC, followed by pattern writing and temperature cycling. The cell is 30 mm in length and 20 mm in width.

To further demonstrate the potential applications of the optical device with photo‐writable and thermo‐responsive patterns, an electrothermal LC cell was prepared. After filling the electrothermal cell with photo‐responsive CLC, the temperature variation under different voltages was recorded, as shown in Figure 6a. Subsequently, a 450 nm blue light with a light intensity of 9 mW/cm^2^ was employed as the writing light source. A “butterfly” pattern was successfully written into the cell via 120 s of 450 nm light irradiation under a photomask. Additionally, a “lantern” pattern was written into a green LC cell via 60 s of 450 nm light irradiation. By applying voltages on the cell, a butterfly pattern in the red cell and a lantern pattern in the green cell appear under 1.5 and 2.0 V, respectively. When the voltage is raised to 2.5 V, the pattern vanishes completely due to the CLC converting to the isotropic state. All of the patterns are capable of reappearing and diminishing reversibly according to the applied voltage. By controlling the light irradiation condition in the photo‐writing, the applied voltage required by the display can be adjusted.

**FIGURE 6:**
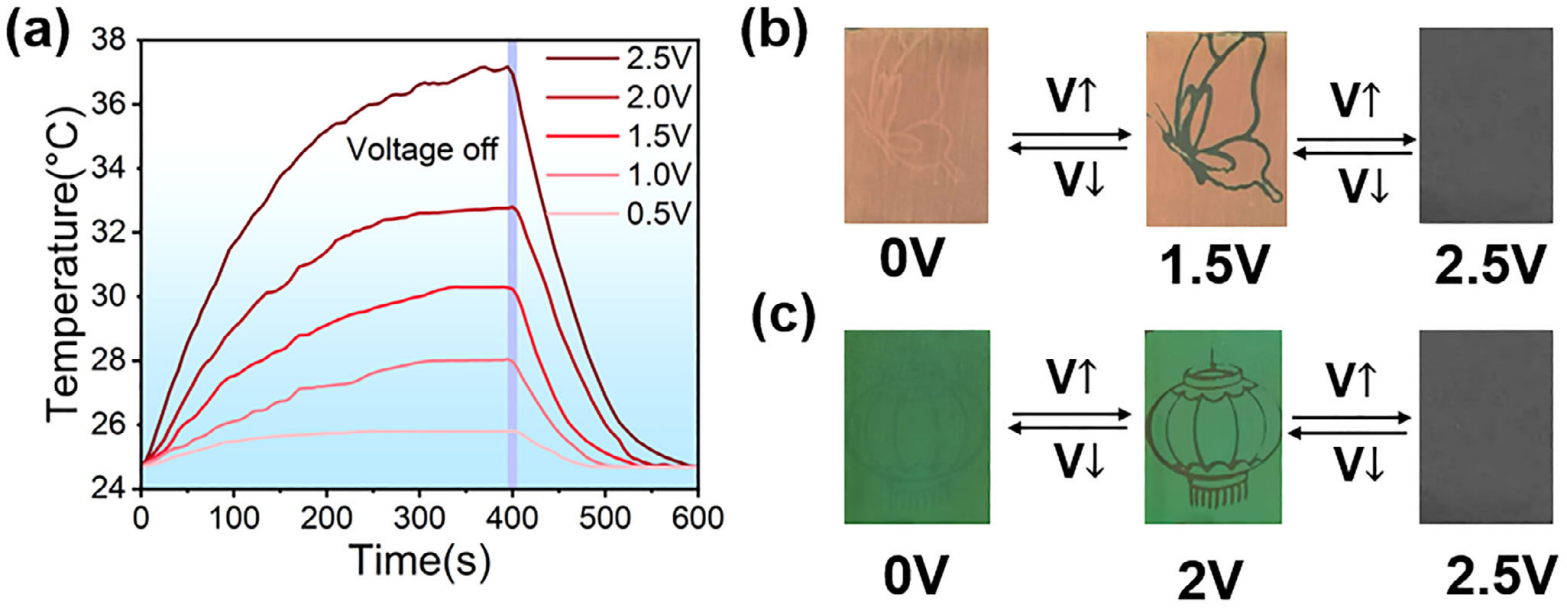
(a) The electrothermal property of the LC cell. Voltage‐driven evolution of the invisible pattern in (b) a red cell and (c) a green cell.

## 3 Conclusion

In summary, a series of bistable photoactive LH‐X molecular switches were judiciously designed and synthesized. The photochemical properties of the LH‐X molecules were investigated in detail. By incorporating the LH‐X molecules into nematic LC, a Photo‐induced isothermal and reversible nematic‐to‐isotropic phase transition of LC was observed. The mechanism of the hydrazone‐driven “order‐disorder” phase transition of LC was further investigated by monitoring the disturbing effect of hydrazone, combined with molecular dynamics simulations. As a critical control, BH‐6, owing to its inherently bent conformation and the minimal structural changes it undergoes upon photoisomerization, exerts only a negligible perturbation on the nematic phase. This observation directly corroborates our central thesis that the substantial linear‐to‐bent shape transformation constitutes the primary driving force for the observed nematic‐to‐isotropic phase transition. Taking advantage of the bistable hydrazone switch, our system enables reversible photoisomerization in both rod‐like and bent conformations, constructing a bidirectional, continuously tunable photoresponsive liquid crystal system. In addition, this bistability allows for accurate determination of light‐induced stepwise phase transition temperatures, providing a new perspective for the design of smart photoresponsive soft materials. By introducing the LH‐X into CLC with structural color, a thermo‐responsive and stepwise information encryption device with photo‐writable patterns was fabricated. This work is hoped to provide a new candidate in the development of light‐driven LC materials and devices with extremely high thermal stability and stepwise tunability.

## 4 Experimental Section

^1^H NMR spectra were recorded on a Bruker 400 MHz spectrometer and a Bruker 600 MHz spectrometer. Chemical shifts (δ) are reported in parts per million (ppm) relative to tetramethylsilane(TMS; δ ^1^H = 0.00, δ ^1^ ^3^C = 0.00) as an internal standard, and residual solvent peaks of CDCl_3_ (δ ^1^H = 7.26, δ ^1^ ^3^C = 77.16) were used as secondary references. The splitting patterns of peaks were designated as follows: s (singlet), d (doublet), t (triplet), m (multiplet), br (broad), or dd (doublet of doublets). Ultraviolet/visible/near‐infrared (UV/Vis/NIR) spectroscopic measurements were recorded using a PerkinElmer Lambda 950 spectrophotometer. Irradiation with light at 365 and 450 nm was performed using an LED lamp (FUV‐6BK, Bangwo Elec. Technologies Co., Ltd., Guangzhou, China). Polarized optical micrographs were obtained using polarized optical microscopy (POM, AxioScope A1 pol; Zeiss, Beijing, China).DSC measurements were performed using a PerkinElmer DSC8000 differential scanning calorimeter at a heating and cooling rate of 10 °C/min. The crystal structure was determined using a single‐crystal X‐ray diffractometer (Oxford Diffraction SuperNova, SCXD).

## Author Contributions

**Shijie Liu**: conceptualization, methodology, investigation, validation, formal analysis, supervision, funding acquisition, project administration, writing – review and editing. **Yuxin Zhou**: methodology, investigation, validation, formal analysis, writing – review and editing. **Junwen Chen**: investigation, validation, formal analysis, writing – review and editing. **Yexin Wang**: investigation, validation, formal analysis, writing – review and editing. **Yiqiu Lv**: investigation, validation, formal analysis, writing – review and editing. **Panjie Wang**: investigation, validation, formal analysis, writing – review and editing. **Wenqi Yu**: investigation, validation, formal analysis, writing – review and editing. **Gege Meng**: investigation, validation, formal analysis, writing – review and editing. **Jiayi Yu**: investigation, validation, formal analysis, writing – review and editing. **Chunhua Lin**: investigation, validation, formal analysis, writing – review and editing. **Guo‐Hua Hu**: investigation, validation, formal analysis, writing – review and editing. **Ruochen Lan**: conceptualization, methodology, investigation, validation, formal analysis, supervision, funding acquisition, project administration, writing – review and editing.

## Conflicts of Interest

The authors declare no conflicts of interest.

## Supporting information




**Supporting File 1**: advs78402‐sup‐0001‐SuppMat.docx.

**Supporting File 2**: advs78402‐sup‐0002‐VideoS1.mp4.

## Data Availability

The data that support the findings of this study are available from the corresponding author upon reasonable request.
