# Supplementary material for: Photo‐Induced Stepwise Liquid Crystal Phase Transition Enabled by Hydrazone Photoswitch
Source: Adv Sci (Weinh). 2026 Oct 22:e78402. Online ahead of print. doi: 10.1002/advs.78402 (PMC13691221; doi:10.1002/advs.78402)
Supplement: Supplementary file 1 [file ADVS-9999-e78402-s001.docx]

Supporting Information

Photo-induced **Stepwise** Liquid Crystal Phase Transition Enabled by Hydrazone Photoswitch

Shijie Liu^1^, Yuxin Zhou^1^, Junwen Chen^1^,Yexin Wang^1^,Yiqiu Lv^1^,Panjie Wang^1^,Wenqi Yu^1^,Gege Meng^1^, Jiayi Yu^1^, Chunhua Lin^1^, Guo-Hua Hu^2^, Ruochen Lan^1*^

**Table of Contents**

**[1. Synthesis and characterization of compounds	3](#_Toc9824)**

**[2. NMR characterization	9](#_Toc8731)**

**[3. UV-vis characterization	26](#_Toc22758)**

**[4. Determination of the thermal isomerization half-life	30](#_Toc8515)**

**[5. Determination of the quantum yield	31](#_Toc478)**

**[6. R and SΦ characterization	37](#_Toc29480)**

**[7. MD Simulation	39](#_Toc25966)**

**[8. DSC characterization	41](#_Toc32221)**

**[9. Reflection characterization	44](#_Toc25020)**

**[10. Supplementary video	44](#_Toc32208)**

**[11. References	44](#_Toc9453)**

# 2.  Synthesis and characterization of compounds

Synthesis of hydrazones **LH-3**

**LH-3①**: Benzoylformic acid (5 g, 33.3 mmol) and DMAP (0.37 g, 30.0 mmol) were added to CH_2_Cl_2_ (100 ml). After the two solids were dissolved, [4-propoxyphenol](https://chem.molaid.com/material/detail?id=30fd5edy75a7106150N0&name=4-%E4%B8%99%E6%B0%A7%E5%9F%BA%E8%8B%AF%E9%85%9A%20%7C%204-propoxyphenol&inchikey=KIIIPQXXLVCCQP-UHFFFAOYSA-N) (4.57 g, 30.0 mmol) was added to them at 0 °C. After 10 minutes of reaction, add DCC (6.19 g, 30.0 mmol) to the mixture. The mixture was stirred while allowed to reach R.T. overnight. After removal of the solvent under vacuum, the product was purified by flash column chromatography (silica gel, EtOAc-PE=1:10→1:6) affording a yellow oil; ^1^H NMR (600 MHz, CDCl_3_) . δ 8.14 – 8.09 (m, 2H), 7.71 (m, 1H), 7.59 – 7.52 (m, 2H), 7.21 – 7.15 (m, 2H), 6.98 – 6.92 (m, 2H), 3.93 (t, J = 6.5 Hz, 2H), 1.82 (m, 2H), 1.05 (t, J = 7.4 Hz, 3H).^13^C NMR (151 MHz, CDCl_3_) δ 185.53, 162.25, 157.50, 143.15, 135.23, 132.36, 130.16, 129.07, 121.99, 115.99, 115.58, 115.26, 69.95, 22.57, 10.53.

**LH-3②**: A mixture consisting of LH-3① (3.01 g, 10.6 mmol) and 4-hydrazinobenzoic acid hydrochloride (1.61 g, 10.6 mmol) in ETOH (30 ml) was heated to 80 °C and then four drops of AcOH were added to the solution.The mixture was stirred for 4 h and then allowed to cool in the refrigerator overnight. The precipitate was collected by filtration yielding a yellow solid; ^1^H NMR (600 MHz, CDCl_3_) δ 12.52 (s, 1H), 8.08 (d, J = 8.6 Hz, 2H), 7.82 – 7.77 (m, 2H), 7.49 – 7.43 (m, 2H), 7.43 – 7.38 (m, 1H), 7.34 – 7.29 (m, 2H), 7.12 – 7.06 (m, 2H), 6.98 – 6.92 (m, 2H), 3.93 (t, J = 6.6 Hz, 2H), 1.82 (hept, J = 6.8 Hz, 2H), 1.05 (t, J = 7.4 Hz, 3H). ^13^C NMR (101 MHz, DMSO) δ 167.51, 161.75, 157.22, 147.45, 143.40, 135.94, 131.52, 131.28, 128.74, 128.65, 124.42, 123.15, 115.49, 114.19, 69.85, 22.48, 10.83.

**LH-3:** A mixture consisting of LH-3② (2.22 g, 5.31 mmol), [4-propoxyphenol](https://chem.molaid.com/material/detail?id=30fd5edy75a7106150N0&name=4-%E4%B8%99%E6%B0%A7%E5%9F%BA%E8%8B%AF%E9%85%9A%20%7C%204-propoxyphenol&inchikey=KIIIPQXXLVCCQP-UHFFFAOYSA-N) (0.93 g, 4.78 mmol), DCC (0.99 g, 4.78 mmol) and DMAP (0.06 g, 0.49 mmol) in CH_2_Cl_2_ was stirred at R.T. for 2 days. After removal of the solvent under vacuum, the product was purified by flash column chromatography (silica gel, EtOAc-PE=1:10→1:6) to afford a yellow solid; ^1^H NMR (600 MHz, CDCl_3_) δ 12.54 (s, 1H), 8.19 – 8.14 (m, 2H), 7.82 – 7.78 (m, 2H), 7.49 – 7.44 (m, 2H), 7.46 – 7.39 (m, 2H), 7.37 – 7.32 (m, 1H), 7.13 – 7.06 (m, 3H), 6.98 – 6.89 (m, 3H), 6.81 – 6.72 (m, 2H), 3.93-3.88 (m, 4H), 1.80 (m, 4H), 1.05 (t, J = 7.4 Hz, 6H). ^13^C NMR (151 MHz, CDCl_3_) δ 165.27, 162.53, 157.40, 156.81, 153.32, 149.38, 147.10, 144.38, 142.97, 135.67, 132.04, 129.84, 128.87, 128.37, 128.18, 123.53, 122.47, 122.16, 115.98, 115.60, 115.27, 115.11, 113.91, 70.22, 70.00, 69.95, 22.69, 22.61, 22.57, 10.54, 10.52.

Synthesis of hydrazones **LH-6**

**LH-6①**: The compound was synthesized following the procedure used for LH-3①, using 4-(hexyloxy)phenol and Benzoylformic acid as the starting materials. The product was purified by flash column chromatography (silica gel, EtOAc-PE=1:10→1:6) to afford a yellow oil. ^1^H NMR (400 MHz, CDCl_3_) δ 8.17 – 8.10 (m, 2H), 7.72 (t, J = 7.4 Hz, 1H), 7.58 (t, J = 7.7 Hz, 2H), 7.20 (d, J = 8.9 Hz, 2H), 6.97 (d, J = 8.6 Hz, 2H), 4.02 – 3.87 (m, 2H), 1.81 (p, J = 6.9 Hz, 2H), 1.49 (p, J = 7.4 Hz, 2H), 1.41 – 1.26 (m, 4H), 0.98 – 0.90 (m, 3H). ^13^C NMR (101 MHz, CDCl_3_) δ 185.51, 162.27, 157.53, 143.18, 135.20, 132.38, 130.12, 129.06, 121.99, 115.25, 68.46, 31.60, 29.22, 25.73, 22.63, 14.07.

**LH-6②**：The compound was synthesized following the procedure used for LH-3②, using LH-6① and 4-hydrazinobenzoic acid hydrochloride as the starting materials. The precipitate was collected by filtration yielding a yellow solid; ^1^H NMR (600 MHz, DMSO) δ 11.92 (s, 1H), 7.94 – 7.88 (m, 2H), 7.82 – 7.77 (m, 2H), 7.50 – 7.38 (m, 5H), 7.31 – 7.24 (m, 2H), 7.04 – 6.97 (m, 2H), 3.98 (t, *J* = 6.5 Hz, 2H), 1.71 (dq, *J* = 8.6, 6.6 Hz, 2H), 1.42 (m, 2H), 1.32 (m, 4H), 0.92 – 0.83 (m, 3H). ^13^C NMR (151 MHz, DMSO) δ 167.70, 167.52, 161.75, 157.21, 147.43, 143.38, 135.93, 131.52, 131.27, 130.14, 129.64, 129.20, 128.75, 128.66, 123.16, 115.48, 114.18, 68.34, 31.47, 29.08, 25.63, 22.54, 14.39.

**LH-6**: The compound was synthesized following the procedure used for LH-3, using LH-6② and [4-(hexyloxy)phenol](https://chem.molaid.com/material/detail?id=310167byf1abea0355N0&name=4-%E5%B7%B1%E6%B0%A7%E5%9F%BA%E8%8B%AF%E9%85%9A%20%7C%204-(hexyloxy)phenol&inchikey=XIIIHRLCKLSYNH-UHFFFAOYSA-N) as the starting materials. The product was purified by flash column chromatography (silica gel, EtOAc-PE=1:10→1:7) affording a yellow solid; ^1^H NMR (600 MHz, CDCl_3_) δ 12.53 (s, 1H), 8.16 (d, *J* = 8.5 Hz, 2H), 7.80 (d, *J* = 7.4 Hz, 2H), 7.46 (t, *J* = 7.5 Hz, 2H), 7.41 (t, *J* = 7.3 Hz, 1H), 7.34 (d, *J* = 8.6 Hz, 2H), 7.10 (t, *J* = 9.0 Hz, 4H), 6.97 – 6.90 (m, 4H), 3.96 (td, *J* = 6.6, 2.9 Hz, 4H), 1.79 (pd, *J* = 6.6, 3.1 Hz, 4H), 1.47 (h, *J* = 6.9 Hz, 4H), 1.35 (q, *J* = 5.2 Hz, 8H), 0.94 – 0.85 (m, 6H). ^13^C NMR (151 MHz, CDCl_3_) δ 165.25, 162.53, 157.42, 156.83, 147.10, 144.38, 142.97, 135.69, 132.05, 129.84, 128.88, 128.38, 128.19, 123.55, 122.48, 122.17, 115.26, 115.09, 113.92, 68.51, 68.44, 31.63, 31.61, 29.28, 29.22, 25.76, 25.74, 22.65, 14.09.

Synthesis of hydrazones **LH-8**

**LH-8①**: The compound was synthesized following the procedure used for LH-3①, using 4-n-octoxyphenol and Benzoylformic acid as the starting materials. The product was purified by flash column chromatography (silica gel, EtOAc-PE=1:10→1:6) to afford a yellow oil. ^1^H NMR (400 MHz, CDCl3) δ 8.14 (d, J = 7.4 Hz, 2H), 7.72 (t, J = 7.4 Hz, 1H), 7.58 (t, J = 7.7 Hz, 2H), 7.25 – 7.15 (m, 2H), 7.01 – 6.92 (m, 2H), 4.03 – 3.87 (m, 2H), 1.82 (p, J = 6.9 Hz, 2H), 1.48 (q, J = 6.7 Hz, 2H), 1.42 – 1.26 (m, 8H), 0.92 (t, J = 6.7 Hz, 3H). ^13^C NMR (101 MHz, CDCl_3_) δ 185.50, 162.23, 157.52, 143.17, 135.18, 132.40, 130.15, 129.05, 121.97, 115.26, 68.74, 31.83, 29.36, 29.25, 26.05, 22.67, 14.11.

**LH-8②**：The compound was synthesized following the procedure used for LH-3②, using LH-8① and 4-hydrazinobenzoic acid hydrochloride as the starting materials. The precipitate was collected by filtration yielding a yellow solid; ^1^H NMR (600 MHz, DMSO) δ 12.01 (s, 1H), 8.00 – 7.92 (m, 2H), 7.87 (d, J = 7.6 Hz, 2H), 7.56 – 7.45 (m, 5H), 7.43 – 7.28 (m, 2H), 7.09 – 7.05 (m, 2H), 4.04 (t, J = 6.6 Hz, 2H), 1.78 (p, J = 7.2 Hz, 2H), 1.47 (q, J = 7.4 Hz, 2H), 1.35-1.25(m, 8H), 0.96 – 0.91 (m, 3H). ^13^C NMR (151 MHz, DMSO) δ 167.51, 161.75, 157.20, 147.45, 143.38, 135.93, 131.52, 131.27, 130.13, 129.66, 128.74, 128.66, 124.37, 123.15, 116.14, 115.78, 115.46, 114.18, 68.32, 68.29, 31.71, 29.32, 29.22, 29.15, 29.12, 26.04, 25.98, 22.56, 14.44.

**LH-8**: The compound was synthesized following the procedure used for LH-3, using LH-8② and 4-n-octoxyphenol as the starting materials. The product was purified by flash column chromatography (silica gel, EtOAc-PE=1:10→1:7) to afford a yellow solid; ^1^H NMR (600 MHz, CDCl_3_) δ 12.45 (s, 1H), 8.08 (d, *J* = 7.8 Hz, 2H), 7.72 (d, *J* = 7.6 Hz, 2H), 7.37 (t, *J* = 7.6 Hz, 2H), 7.35 – 7.31 (m, 1H), 7.29 – 7.24 (m, 2H), 7.08 – 6.99 (m, 4H), 6.85 (m, 4H), 3.87 (t, *J* = 6.6 Hz, 4H), 1.70 (h, *J* = 6.4 Hz, 4H), 1.45 – 1.20 (m, 20H), 0.81 (t, *J* = 6.9 Hz, 6H). ^13^C NMR (151 MHz, CDCl_3_) δ 165.25, 162.54, 157.41, 156.82, 147.09, 144.36, 142.95, 135.67, 132.04, 129.83, 128.87, 128.37, 128.18, 124.00, 123.55, 122.47, 122.15, 115.26, 115.09, 113.91, 68.51, 68.45,. 31.53, 31.46, 30.33, 30.21, 30.15, 29.72, 29.39, 29.37, 29.30, 29.27, 29.24, 26.07, 26.05, 22.72, 22.68, 14.13.

Synthesis of hydrazones **LH-12**

**LH-12①**: The compound was synthesized following the procedure used for LH-3①, using 4-dodecyloxyphenol and Benzoylformic acid as the starting materials. The product was purified by flash column chromatography (silica gel, EtOAc-PE=1:10→1:6) to afford a yellow oil. ^1^H NMR (600 MHz, CDCl_3_) δ 8.13 – 8.09 (m, 2H), 7.73 – 7.67 (m, 1H), 7.56 (t, *J* = 7.8 Hz, 2H), 7.20 – 7.15 (m, 2H), 6.97 – 6.91 (m, 2H), 3.96 (t, *J* = 6.6 Hz, 2H), 1.79 (dt, *J* = 14.9, 6.7 Hz, 2H), 1.52 – 1.40 (m, 2H), 1.39 – 1.25 (m, 12H), 0.88 (t, *J* = 6.9 Hz, 3H). ^13^C NMR (151 MHz, CDCl_3_) δ 185.51, 162.23, 157.50, 143.13, 135.21, 132.38, 130.17, 129.06, 121.97, 115.59, 68.72, 31.94, 29.37, 29.24, 26.07, 26.04, 22.71, 14.14.

**LH-12②**：The compound was synthesized following the procedure used for LH-3②, using LH-12① and 4-hydrazinobenzoic acid hydrochloride as the starting materials. The precipitate was collected by filtration yielding a yellow solid; ^1^H NMR (600 MHz, CDCl3) δ 12.50 (s, 1H), 8.08 (d, J = 8.4 Hz, 2H), 7.81 – 7.76 (m, 2H), 7.45 (dd, J = 8.3, 6.5 Hz, 2H), 7.41 (dd, J = 8.5, 6.1 Hz, 1H), 7.31 (d, J = 8.4 Hz, 2H), 7.11 – 7.06 (m, 2H), 6.97 – 6.91 (m, 2H), 3.96 (t, J = 6.5 Hz, 2H), 1.83 – 1.72 (m, 2H), 1.45 (p, J = 7.3 Hz, 2H), 1.28 (m, 12H), 0.88 (t, J = 7.0 Hz, 3H).^13^C NMR (151 MHz, DMSO) δ 167.51,161.75 157.20, 147.44, 143.38, 135.92, 131.52, 131.28, 128.75, 128.66, 124.39, 123.15, 116.13, 115.78, 115.47, 114.18, 68.32, 31.77, 29.51, 29.48, 29.46, 29.23, 29.18, 29.10, 25.95, 22.57, 14.43.

**LH-12**: The compound was synthesized following the procedure used for LH-3, using LH-12② and 4-dodecyloxyphenol as the starting materials. The product was purified by flash column chromatography (silica gel, EtOAc-PE=1:10→1:7) to afford a yellow solid;^1^H NMR (600 MHz, CDCl_3_) δ 12.52 (s, 1H), 8.16 (d, *J* = 8.5 Hz, 2H), 7.80 (d, *J* = 7.6 Hz, 2H), 7.46 (t, *J* = 7.5 Hz, 2H), 7.42 (m, 1H), 7.34 (d, *J* = 8.5 Hz, 2H), 7.10 (t, *J* = 9.2 Hz, 4H), 6.97 – 6.89 (m, 4H), 3.96 (td, *J* = 6.5, 2.9 Hz, 4H), 1.78 (m, 4H), 1.45 (p, *J* = 7.5 Hz, 4H), 1.38 – 1.21 (m, 24H), 0.88 (t, *J* = 6.9 Hz, 6H).^13^C NMR (151 MHz, CDCl_3_) δ 165.25, 162.54, 157.41, 156.82, 147.09, 144.36, 142.95, 135.67, 132.04, 129.83, 128.87, 128.37, 128.18, 123.55, 122.46, 122.15, 115.26, 115.09, 115.04, 113.90, 68.51, 68.45, 31.94, 29.69, 29.66, 29.62, 29.60, 29.42, 29.40, 29.37, 29.30, 29.24, 26.06, 26.04, 22.72, 14.15.

Synthesis of hydrazones **BH-6**

**BH-6①**: A mixture consisting of methyl 2-(4-hydroxyphenyl)-2-oxoacetate (2 g, 11.1 mmol), p-hexyloxybenzoic acid (2.74 g, 12.23 mmol), DCC (2.29 g, 11.1 mmol) and DMAP (0.14 g, 0.111 mmol) in CH_2_Cl_2_ was stirred at R.T. for 2 days. After removal of the solvent under vacuum, the product was purified by flash column chromatography (silica gel, EtOAc-PE=1:2) affording a white powder; ^1^H NMR (400 MHz, CDCl_3_) δ 8.15 (m, 4H), 7.41 (d, *J* = 8.4 Hz, 2H), 7.01 (d, *J* = 8.6 Hz, 2H), 4.08 (t, *J* = 6.5 Hz, 2H), 4.01 (s, 3H), 1.85 (p, *J* = 6.8 Hz, 2H), 1.51 (t, *J* = 7.4 Hz, 2H), 1.38 (h, *J* = 4.0 Hz, 2H), 1.33 (s, 2H), 0.98 – 0.90 (m, 3H). ^13^C NMR (101 MHz, CDCl_3_) δ 184.65, 164.06, 163.94, 163.78, 156.43, 132.47, 131.88, 129.86, 122.43, 120.74, 114.47, 68.42, 52.83, 31.54, 29.04, 25.65, 22.58, 14.01.

**BH-6②**: A mixture consisting of BH-6① (1.66 g, 4.45 mmol) and 4-hydrazinobenzoic acid hydrochloride (0.68 g, 4.45 mmol) in ETOH (20ml) was heated to 80 °C and then four drops of AcOH were added to the solution. The mixture was stirred for 4 h and then allowed to cool in the refrigerator overnight. The precipitate was collected by filtration yielding a yellow solid;^1^H NMR (600 MHz, DMSO) δ 11.67 (s, 1H), 8.12 – 8.06 (m, 2H), 7.93 – 7.87 (m, 2H), 7.76 – 7.71 (m, 2H), 7.45 – 7.39 (m, 2H), 7.34 – 7.29 (m, 2H), 7.16 – 7.10 (m, 2H), 4.10 (t, *J* = 6.5 Hz, 2H), 3.92 (s, 3H), 1.79 – 1.72 (m, 2H), 1.44 (p, *J* = 7.2 Hz, 2H), 1.32 (m, 4H), 0.92 – 0.83 (m, 3H). ^13^C NMR (151 MHz, DMSO) δ 167.54, 164.66, 163.75, 163.53, 151.17, 147.69, 132.73, 132.55, 131.88, 131.52, 129.23, 123.88, 123.46, 122.30, 121.14, 115.18, 113.84, 68.49, 52.94, 31.44, 28.92, 25.57, 22.53, 14.38.

**BH-6:** A mixture consisting of BH-6② (1.60 g, 3.17 mmol), [4-(hexyloxy)phenol](https://chem.molaid.com/material/detail?id=310167byf1abea0355N0&name=4-%E5%B7%B1%E6%B0%A7%E5%9F%BA%E8%8B%AF%E9%85%9A%20%7C%204-(hexyloxy)phenol&inchikey=XIIIHRLCKLSYNH-UHFFFAOYSA-N) (0.56 g, 2.88 mmol), DCC (0.59 g, 2.88 mmol) and DMAP (0.04 g, 0.3 mmol) in CH_2_Cl_2_ was stirred at R.T. for 2 days. After removal of the solvent under vacuum, the product was purified by flash column chromatography (silica gel, EtOAc-PE=1:4) affording a yellow solid; ^1^H NMR (600 MHz, DMSO) δ 11.65 (s, 1H), 8.13 – 8.01 (m, 4H), 7.78 – 7.73 (m, 2H), 7.51 (d, J = 8.8 Hz, 2H), 7.35 – 7.31 (m, 2H), 7.19 – 7.11 (m, 4H), 7.01 – 6.94 (m, 2H), 4.18 – 4.07 (m, 2H), 3.98 (t, J = 6.5 Hz, 2H), 3.94 (s, 3H), 1.80 – 1.61 (m, 4H), 1.43 (q, J = 7.8 Hz, 4H), 1.37 – 1.27 (m, 12H), 0.92 – 0.83 (m, 6H). ^13^C NMR (151 MHz, CDCl_3_) δ 165.31, 164.88, 163.89, 163.64, 156.80, 151.09, 147.30, 144.39, 133.32, 132.44, 132.35, 132.04, 129.90, 129.71, 123.19, 122.49, 122.47, 121.42, 115.08, 114.46, 114.34, 113.73, 68.44, 68.37, 52.19, 31.61, 31.57, 29.27, 29.08, 25.74, 25.68, 22.64, 22.61, 14.07, 14.06.

# 3. NMR characterization

**Figure S1.** ^1^H NMR spectrum of **LH-3①** in CDCl_3_ at 294 K.

**Figure S2.** ^13^C NMR spectrum of **LH-3①** in CDCl_3_ at 294 K.

**Figure S3.** ^1^H NMR spectrum of **LH-3②** in CDCl_3_ at 294 K.

**Figure S4.** ^13^C NMR spectrum of **LH-3②** in Dmso at 294 K.

**Figure S5.** ^1^H NMR spectrum of **LH-3** in CDCl_3_ at 294 K.

**Figure S6.** ^13^C NMR spectrum of **LH-3** in CDCl_3_ at 294 K.

**Figure S7.** ^1^H NMR spectrum of **LH-6①** in CDCl_3_ at 294 K.

**Figure S8.** ^13^C NMR spectrum of **LH-6①** in CDCl_3_ at 294 K.

**Figure S9.** ^1^H NMR spectrum of **LH-6②** in DMSO at 294 K.

**Figure S10.** ^13^C NMR spectrum of **LH-6②** in DMSO at 294 K.

**Figure S11.** ^1^H NMR spectrum of **LH-6** in CDCl_3_ at 294 K.

**Figure S12.** ^13^C NMR spectrum of **LH-6** in CDCl_3_ at 294 K.

**Figure S13.** ^1^H NMR spectrum of **LH-8①** in CDCl_3_ at 294 K.

**Figure S14.** ^13^C NMR spectrum of **LH-8①** in CDCl_3_ at 294 K.

**Figure S15.** ^1^H NMR spectrum of **LH-8①** in DMSO at 294 K.

**Figure S16.** ^13^C NMR spectrum of **LH-8②** in DMSO at 294 K.

**Figure S17.** ^1^H NMR spectrum of **LH-8** in CDCl_3_ at 294 K.

**Figure S18.** ^13^C NMR spectrum of **LH-8** in CDCl_3_ at 294 K.

**Figure S19.** ^1^H NMR spectrum of **LH-12①** in CDCl_3_ at 294 K.

**Figure S20.** ^13^C NMR spectrum of **LH-12①** in CDCl_3_ at 294 K.

**Figure S21.** ^1^H NMR spectrum of **LH-12②** in CDCl_3_ at 294 K.

**Figure S22.** ^13^C NMR spectrum of **LH-12②** in DMSO at 294 K.

**Figure S23.** ^1^H NMR spectrum of **LH-12** in CDCl_3_ at 294 K.

**Figure S24.** ^13^C NMR spectrum of **LH-12** in CDCl_3_ at 294 K.

**Figure S25.** ^1^H NMR spectrum of **BH-6①** in CDCl_3_ at 294 K.

**Figure S26.** ^13^C NMR spectrum of **BH-6①** in CDCl_3_ at 294 K.

**Figure S27.** ^1^H NMR spectrum of **BH-6②** in DMSO at 294 K.

**Figure S28.** ^13^C NMR spectrum of **BH-6②** in DMSO at 294 K.

**Figure S29.** ^1^H NMR spectrum of **BH-6** in CDCl_3_ at 294 K.

**Figure S30.** ^13^C NMR spectrum of **BH-6** in CDCl_3_ at 294 K.

**Figure S31.** ^1^H NMR spectra (Chloroform-d, 294 K) of LH-3 (A) before, and after (B) 450 nm followed by (C) 340 nm photo irradiation to reach the PSS.

**Figure S32.** ^1^H NMR spectra (Chloroform-d, 294 K) of LH-8 (A) before, and after (B) 450 nm followed by (C) 340 nm photo irradiation to reach the PSS.

**Figure S33.** ^1^H NMR spectra (Chloroform-d, 294 K) of LH-12 (A) before, and after (B) 450 nm followed by (C) 340 nm photo irradiation to reach the PSS.

**Figure S34.** ^1^H NMR spectra (Chloroform-d, 294 K) of BH-6 (A) before, and after (B) 450 nm followed by (C) 340 nm photo irradiation to reach the PSS.

# 4. UV-vis characterization

**Figure S35.** UV-Vis absorption spectra of **LH-3** (10 μM)in THF.

**Figure S36.** Cycling test of the photo-induced UV-vis changes of **LH-3.**

**Figure S37.** UV-Vis absorption spectra of **LH-8** (10 μM)in THF.

**Figure S38.** Cycling test of the photo-induced UV-vis changes of **LH-8**.

**Figure S39.** UV-Vis absorption spectra of **LH-12** (10μM)in THF.

**Figure S40.** Cycling test of the photo-induced UV-vis changes of **LH-12.**

**Figure S41.** UV-Vis absorption spectra of **BH-6** (10 μM)in THF.

**Figure S42.** Cycling test of the photo-induced UV-vis changes of **BH-6.**

# 5. Determination of the thermal isomerization half-life

The thermal half-life of LH-X and BH-6 was determined by following reported procedures.1wt% sample in 5CB was prepared, and irradiated with 450 nm light until the photostationary statewas reached. The sample was heated to 120 °C for 5 hours and the change in concentration of the E-isomer measured. This change in isomeric ratio was used to determine the rate of decay (equation 1), which enabled the calculation of the energy barrier for relaxation from E → Z using the Eyring equation (equation 2). With the activation barrier in hand the Arrhenius equation (equation 3) was used to extrapolate the thermal half-life at room temperature.

k=$\frac{\text{ln}\text{(}\frac{\text{\%}\text{Eo}}{\text{\%}\text{E}}\text{)}}{\text{t}}$	(1)

Where *k* indicates the rate constant, *%E0* indicates the initial percentage of the E isomer, *%E* indicates the percentage of the E isomer after incubation and t is the time in seconds.

ΔG^‡^=8.314∙T∙[23.760+ln($\frac{\text{T}}{\text{k}}$)]	(2)

Where *ΔG^‡^* indicates the energy barrier to relaxation in J mol^-1^ and T is the incubation temperature.

ln($\frac{\text{k}_{\text{2}}}{\text{k}_{\text{1}}}$)=$\frac{\text{Δ}\text{G}}{\text{R}}$($\frac{\text{1}}{\text{T}_{\text{1}}}\text{-}\frac{\text{1}}{\text{T}_{\text{2}}}$)	(3)

Where *k_2_* indicates the rate at the room temperature, *k_1_* indicates the rate at the incubation temperature, *R* indicates the ideal gas constant in J K^-1^ mol^-1^, *T_2_* is 298 K, and *T_1_* indicates the incubation temperature in kelvin.

**Table S1.**Kinetic Data for the Thermal Isomerization of Compounds LH-X and BH-6 in 5CB at 298 K.

| **Hydrazones^a^** | ***k_2_*^b^(s^-1^)** | **ΔG^≠c^(KJ mol^-1^)** | **τ_1/2_^d^(years)** |
| --- | --- | --- | --- |
| LH-3 | 1.4×10^-11^ | 135.51 | 1591 |
| LH-6 | 7.52×10^-10^ | 125 | 29.63 |
| LH-8 | 5.6×10^-10^ | 128.76 | 39.79 |
| LH-12 | 8.216×10^-12^ | 136.89 | 2712 |
| BH-6 | 1.2×10^-11^ | 135.78 | 1857 |

^a^Thermal relaxation occurs from E → Z.^b^The first-order rate constants at 298 K were calculated using the Arrhenius equation.^c^The Gibbs energy of activation was determinedusing the Eyring equation.^d^Half-life at 298 K.

# 6. Determination of the quantum yield

The quantum yield was measured according to the experimental procedures reported in the references^[1,2]^.The observed rate constants were determined by monitoring the time-dependent absorbance changes of 10 μM solutions of LH-X and BH-6 in tetrahydrofuran upon irradiation at 450 nm, followed by fitting the data to Equation 4 (Figures S43–S52). Given the negligible thermal relaxation due to the exceptionally long half-life of the compounds (vide supra). The observed rate constant was used in equation 5 to determine *Φ_Z→E_*. The same procedure was conducted under 340 nm irradiation to obtain *Φ_E→Z_*.

$\text{y}\text{=}\text{y}_{\text{0}}\text{+}\text{A}_{\text{0}}\text{e}^{\text{R}_{\text{0}}\text{x}}$	(4)

$$\text{Φ}_{\text{A}\text{→}\text{B}}\text{=}\frac{\text{k}_{\text{A}\text{→}\text{B}}\text{V}}{\text{I}_{\text{0}}\text{ε}_{\text{A}}\text{l}\ln\text{10}}\text{ (5)}$$

Where *Φ* indicates the quantum yield, *k_A→B_* indicates the R0 value from equation 4, V indicates the volume of solution, *I_0_* indicates the molar flux of light, *ε_A_* indicates the molar absorbtivity and *l* indicates the path length.

**Table S2.**Photophysical Data for Compounds LH-X and BH-6 in THF

| **Hydrazone** | **λmax(nm)/ε** | | **Φ** | | **PSS@λirr(nm)** | |
| --- | --- | --- | --- | --- | --- | --- |
|  | **Z** | **E** | **Z→E** | **E→Z** |  |  |
| LH-3 | 375/43318 | 344/41599 | 29.67% | 19.40% | 88%ofE@450 | 85%ofZ@340 |
| LH-6 | 375/43417 | 344/35184 | 29.82% | 8.43% | 100%ofE@450 | 83%ofZ@340 |
| LH-8 | 374/44228 | 349/36369 | 30.55% | 9.56% | 100%ofE@450 | 79%ofZ@340 |
| LH-12 | 372/37983 | 350/32781 | 28.38% | 8.12% | 100%ofE@450 | 80%ofZ@340 |
| BH-6 | 372/46253 | 345/38420 | 11.91% | 1.63% | 96%ofE@450 | 91%ofZ@340 |

**Figure S43.**Kinetics of the Z→E photoisomerization of LH-3 (1 × 10⁻⁵ M in THF) upon irradiation at 450 nm. The plot shows the absorbance of the E-isomer at 340 nm as a function of irradiation time.

**Figure S44.**Kinetics of the E→Z photoisomerization of LH-3 (1 × 10⁻⁵ M in THF) upon irradiation at 340 nm. The plot shows the absorbance of the Z-isomer at 340 nm as a function of irradiation time.

**Figure S45.**Kinetics of the Z→E photoisomerization of LH-6 (1 × 10⁻⁵ M in THF) upon irradiation at 450 nm. The plot shows the absorbance of the E-isomer at 340 nm as a function of irradiation time.

**Figure S46.**Kinetics of the E→Z photoisomerization of LH-6 (1 × 10⁻⁵ M in THF) upon irradiation at 340 nm. The plot shows the absorbance of the Z-isomer at 340 nm as a function of irradiation time.

**Figure S47.**Kinetics of the Z→E photoisomerization of LH-8 (1 × 10⁻⁵ M in THF) upon irradiation at 450 nm. The plot shows the absorbance of the E-isomer at 340 nm as a function of irradiation time.

**Figure S48.**Kinetics of the E→Z photoisomerization of LH-8 (1 × 10⁻⁵ M in THF) upon irradiation at 340 nm. The plot shows the absorbance of the Z-isomer at 340 nm as a function of irradiation time.

**Figure S49.**Kinetics of the Z→E photoisomerization of LH-12 (1 × 10⁻⁵ M in THF) upon irradiation at 450 nm. The plot shows the absorbance of the E-isomer at 340 nm as a function of irradiation time.

**Figure S50.**Kinetics of the E→Z photoisomerization of LH-12 (1 × 10⁻⁵ M in THF) upon irradiation at 340 nm. The plot shows the absorbance of the Z-isomer at 340 nm as a function of irradiation time.

**Figure S51.** Kinetics of the Z→E photoisomerization of BH-6 (1 × 10⁻⁵ M in THF) upon irradiation at 450 nm. The plot shows the absorbance of the E-isomer at 340 nm as a function of irradiation time.

**Figure S52.**Kinetics of the E→Z photoisomerization of BH-6 (1 × 10⁻⁵ M in THF) upon irradiation at 340 nm. The plot shows the absorbance of the Z-isomer at 340 nm as a function of irradiation time.

# 7. R and S_Φ_ characterization

**Figure S53.** (a) Linearly polarized absorption spectra of hydrazone LH-3 under different states . (b) Linearly polarized absorption spectra of DR1 in LH-3 doped 5CB under different states.

**Figure S54.** (a) Linearly polarized absorption spectra of hydrazone LH-8 under different states . (b) Linearly polarized absorption spectra of DR1 in LH-8 doped 5CB under different states.

**Figure S55.** (a) Linearly polarized absorption spectra of hydrazone LH-12 under different states . (b) Linearly polarized absorption spectra of DR1 in LH-12 doped 5CB under different states.

**Figure S56.** (a) Linearly polarized absorption spectra of hydrazone BH-6 under different states . (b) Linearly polarized absorption spectra of DR1 in BH-6 doped 5CB under different states.

# 8. MD Simulation

Liquid crystal molecules were modeled, with two structures: the original structure before light exposure and the isomerized structure after light exposure. Using the Packmol program, 300 molecular chains were randomly placed into a 10×10×10 nm box for each structure, resulting in two separate systems. The OPLS force field combined with RESP charges was selected for the simulations.

All simulation procedures were performed using the open-source Large-scale Atomic/Molecular Massively Parallel Simulator (LAMMPS) platform. First, energy minimization was carried out on the models using the conjugate gradient (CG) algorithm to obtain the geometrically optimal configuration. The energy tolerance and force tolerance convergence criteria were set to 1×10⁻¹⁵ kcal/mol and 1×10⁻¹⁵ kcal/mol, respectively, with a maximum iteration step of 20,000 (dynamics time steps) and a maximum number of function evaluations of 1,000,000.

Subsequently, initial velocities were assigned to all atoms following a Gaussian distribution at 300 K. The cutoff distance for Lennard-Jones interactions was set to 1.2 nm, and the long-range electrostatic interactions were calculated using the PPPM solver. For non‑bonded interaction truncation, special_bonds settings were: 1‑2 and 1‑3 interactions set to 0, and 1‑4 interactions set to 0.5 (for both LJ and Coulomb terms). The time step was 1 fs (global setting).

The overall process followed an annealing cycle: room temperature → heating → high temperature → cooling → room temperature.

① NPT ensemble equilibration at 1 atm and 300 K for 1 ns.

② NPT ensemble at 1 atm, temperature ramped to 500 K over 1 ns.

③ NPT ensemble at 1 atm and 500 K for 2 ns.

④ NPT ensemble at 1 atm, temperature ramped down to 300 K over 1 ns.

⑤ NPT ensemble equilibration at 1 atm and 300 K for 1 ns.

The system thus reached an equilibrium state. During this process, structural evolution was recorded, and parameters such as radius of gyration (Rg), radial distribution function (RDF), and interaction energy were calculated over the last 1 ns.

**Figure S57.** The S_2_ of the liquid crystal molecules changes with different states.

# 9. DSC characterization

**Figure S58.** T_NI_ of (a) LH-3 doped 5CB, (b) LH-3 doped 7CB and (c) LH-3 doped 6OCB at different states.

**Figure S59.** T_NI_ of (a) LH-8 doped 5CB, (b) LH-8 doped 7CB and (c) LH-8 doped 6OCB at different states.

**Figure S60.** T_NI_ of (a) LH-12 doped 5CB, (b) LH-12 doped 7CB and (c) LH-12 doped 6OCB at different states.

**Figure S61.** T_NI_ of (a) BH-6 doped 5CB, (b) BH-6 doped 7CB and (c) BH-6 doped 6OCB at different states.

**Table S3.** T_NI_ of different molecules at different States.

|  | **LH-3**  **Initial**  **T_NI_(°C)** | **LH-3**  **PSS_450_**  **T_NI_(°C)** | **LH-3**  **PSS_340_**  **T_NI_(°C)** | **LH-6**  **Initial**  **T_NI_(°C)** | **LH-6**  **PSS_450_**  **T_NI_(°C)** | **LH-6**  **PSS_340_**  **T_NI_(°C)** | **LH-8**  **Initial**  **T_NI_(°C)** |
| --- | --- | --- | --- | --- | --- | --- | --- |
| 5CB | 33.2±0.25 | 27.95±0.15 | 31.6±0.22 | 34.07±0.17 | 28.00±0.26 | 32.01±0.25 | 32.64±0.16 |
| 7CB | 40.35±0.21 | 36.36±0.26 | 39.56±0.12 | 41.11±0.15 | 36.45±0.15 | 40.11±0.22 | 40.89±0.12 |
| 6OCB | 74.27±0.13 | 70.08±0.18 | 72.17±0.32 | 74.57±0.15 | 70.11±0.21 | 72.11±0.28 | 73.36±0.19 |

**Table S3 (continued).**

| **LH-8**  **PSS_450_**  **T_NI_(°C)** | **LH-8**  **PSS_340_**  **T_NI_(°C)** | **LH-12**  **Initial**  **T_NI_(°C)** | **LH-12**  **PSS_450_**  **T_NI_(°C)** | **LH-12**  **PSS_340_**  **T_NI_(°C)** | **BH-6**  **Initial**  **T_NI_(°C)** | **BH-6**  **PSS_450_**  **T_NI_(°C)** | **BH-6**  **PSS_340_**  **T_NI_(°C)** |
| --- | --- | --- | --- | --- | --- | --- | --- |
| 28.31±0.15 | 30.00±0.25 | 33.91±0.15 | 29.32±0.15 | 31.67±0.26 | 32.88±0.15 | 32.88±0.15 | 32.88±0.15 |
| 36.67±0.21 | 39.18±0.18 | 41.64±0.18 | 37.49±0.23 | 40.35±0.22 | 39.57±0.20 | 39.57±0.20 | 39.57±0.20 |
| 70.64±0.18 | 72.73±0.21 | 75.08±0.10 | 73.74±0.17 | 74.46±0.10 | 72.52±0.35 | 72.52±0.35 | 72.52±0.35 |

**Table S4.** ΔT for different molecules at different states.

|  | **LH-3**  **PSS450**  **(ΔT°C)** | **LH-3**  **PSS3400**  **(ΔT°C)** | **LH-6**  **PSS450**  **(ΔT°C)** | **LH-6**  **PSS340**  **(ΔT°C)** | **LH-8**  **PSS450**  **(ΔT°C)** | **LH-8**  **PSS340**  **(ΔT°C)** | **LH-12**  **PSS450**  **(ΔT°C)** | **LH-12**  **PSS340**  **(ΔT°C)** | **BH-6**  **PSS450**  **(ΔT°C)** | **BH-6**  **PSS340**  **(ΔT°C)** |
| --- | --- | --- | --- | --- | --- | --- | --- | --- | --- | --- |
| 5CB | 5.25±0.40 | 3.65±0.37 | 6.07±0.43 | 4.01±0.51 | 4.33±0.31 | 1.69±0.40 | 4.59±0.40 | 2.35±0.30 | 0±0.30 | 0±0.30 |
| 7CB | 3.99±0.47 | 3.20±0.38 | 4.66±0.30 | 3.66±0.37 | 4.22±0.33 | 2.51±0.39 | 4.15±0.41 | 2.86±0.45 | 0±0.40 | 0±0.40 |
| 6OCB | 4.19±0.31 | 2.09±0.40 | 4.46±0.36 | 2.00±0.49 | 2.72±0.37 | 2.09±0.39 | 1.34±0.27 | 0.72±0.27 | 0±0.70 | 0±0.70 |

**Figure S62.**Clearing temperature measured at various storage times (0–24 h) in the dark after the sample was irradiated at 9 mW/cm² for 60 s.

**Figure S63.**Time-dependent temperature profiles of the blank LC cell under irradiation at various intensities (2, 3, 5, 7, and 9 mW/cm²) at room temperature.

# 10. Reflection characterization

**Figure S64.** Reflection changes of CLC with varied S5011 concentrations in different states.

# 11. Supplementary video

**Supplementary video.**Voltage-controlled reversible patterning in a green cholesteric liquid crystal (CLC) cell. The video was recorded with a smartphone and is played at 10× speed. A lantern pattern was written into the green CLC cell using 450 nm light. Upon applying an external voltage of 2.0 V, the pre-written lantern pattern became visible. When the voltage was increased to 2.5 V, the CLC transitioned to the isotropic state and the pattern disappeared completely. After the applied voltage was removed, the lantern pattern recovered immediately.

# 12. References

[1]	B. Shao, H. Qian, Q. Li, I. Aprahamian, *J. Am. Chem. Soc.* **2019**, *141*, 8364.

[2]	M. J. Moran, M. Magrini, D. M. Walba, I. Aprahamian, *J. Am. Chem. Soc.* **2018**, *140*, 13623.
